# Implementation of a non-randomized controlled trial of yoga-based intervention to reduce behavioural issues in early adolescent school-going children in Sri Lanka

**DOI:** 10.1186/s12992-022-00819-3

**Published:** 2022-03-05

**Authors:** Josephine Thirumagal Sivashankar, Rajendra Surenthirakumaran, Shannon Doherty, Nalini Sathiakumar

**Affiliations:** 1 Office of the Regional Director of Health Services, Jaffna, Sri Lanka; 2 Department of Family and Community Medicine, University of Jaffna, Jaffna, Sri Lanka; 3 School of Allied Health, Anglia Ruskin University, Cambridge, UK; 4 Department of Epidemiology, School of Public Health, University of Alabama at Birmingham, Birmingham, USA

**Keywords:** Adolescents, Teenagers, Post-conflict, Behavioural problems, Yoga-based intervention

## Abstract

**Background:**

Adolescence can be difficult to navigate and the post-conflict environment in Jaffna Province, Sri Lanka compounds existing issues for adolescents. Conduct problems, hyperactivity along with emotional problems are challenges faced by adolescents, particularly in fragile, post-conflict settings. This study was a non-randomized controlled trial carried out in 4 educational zones over 6 months. The study implemented a yoga-based intervention package: two types of slow breathing for 5-6 min, Surya-namaskaram for 6-8 min, and mindfulness meditation for 5-6 min. Pre/post quantitative assessments were conducted with intervention and control groups. A focus group was conducted with the intervention group. The aim was to evaluate. Effectiveness of implementing a yoga-based intervention package in grade 8 school children (early adolescents) to address behavioural problems.

**Results:**

Paired t-test and independent t-tests were completed for both arms using statistical product and service solutions (SPSS21). Parents’ assessments of emotional issues reduced for the intervention group (*n* = 584) [*t*(584) = 11.41, *p* = 0.001] along with reduction of the total difficulty score [*t*(584) = 28.12, *p* = 0.001]. Teachers’ assessments indicated prosocial scores improved in the intervention group [*t*(584) = − 28.5, *p* = 0.001]. Students’ self-assessments in the intervention group indicate a reduction in emotional problems [*t*(584) = 6.4, *p* = 0.001], and reduction in problems with peers [*t*(584) = 14.4, *p* = 0.001]. Within the control group (*n* = 499), teachers’ assessments indicated emotional problems increased [*t*(499) = − 9.5, *p* = 0.001] and prosocial scores reduced [*t*(499) = 13.5, *p* = 0.001]. Students’ self-assessments in the control group indicated emotional problems increased [*t*(499) = − 27.1, *p* = 0.001]. A comparison of post-test scores revealed a statistically significant difference between groups. Focus group results indicate students felt the intervention had an overall positive effect on school achievements, family dynamics and individual health.

**Conclusions:**

This yoga-based intervention package appeared to be effective in reducing both externalizing and internalizing symptoms in adolescents. Practicing Surya-namaskaram, breathing control techniques and mindfulness meditation significantly reduced both externalizing symptoms (conduct problems and hyperactivity) as well as internalizing symptom (emotional problem and peer problems). It is recommended this intervention be scaled up across Sri Lanka and other similar post-conflict regions.

## Background

The adolescent period marks a transitional phase between childhood and adulthood where a dependent vulnerable child is transformed into a strong independent adult. The World Health Organization (WHO) defines adolescents as the age group between 10 and 19 years [1]. The United Nations defines “youth” as persons aged between 15 and 24 years and “young people” as persons aged between 10 and 24 years [1]. This characterizes the time period starting from puberty to an economically independent adult [2]. Within this age group adolescents can struggle to cope with physical, psychological, and emotional development. They are often subject to peer pressure, school achievement pressure, parental expectations, social affirmation, and bombarded by technology. Adolescents engaging in these struggles run the risk of being labelled troublemakers and uncontrollable by school teachers, and unmanageable and irresponsible by their parents and the society. These issues can also be compounded within a post-conflict situation where adolescents may have been exposed to traumatic events which can lead to worsening of emotional and behavioural problems [1].

The WHO’s “Health for the world’s adolescents” report indicates that the predominant cause of illness in boys and girls aged 10- 19 years is depression [2]. Globally, the top 3 causes of adolescent death are road traffic accidents, Human Immuno Defficiency Virus/Aquired Immuno Deficiency Syndrome (HIV/AIDS) and suicide [2–5]. The Youth Risk Behaviour Surveillance study carried out by the Centre for Disease Control (CDC) revealed 7 major risk behaviours for adolescents [6]. Risk factors included: un-intentional injuries, violence, tobacco use, alcohol and other drug use, unhealthy sexual behaviours leading to unwanted pregnancies and sexually transmitted diseases (STDs), inadequate diet, poor physical activity [6].

Behavioural disorders in adolescents can be classified as follows; anxiety disorders, severe depression, bipolar disorders, attention –deficit/hyperactivity disorders, learning disorders, conduct disorders, and adjustment problems of adolescents with themselves, with parents, with their school discipline and the community [4, 5]. Behavioural problems among school-going adolescents are multi-factorial in nature and can go unnoticed by parents and teachers, and are therefore early intervention is not engaged with. When behavioural problems of adolescents go unidentified or are left untreated it may lead to several negative health consequences such as obesity, high blood pressure, stroke, unhealthy lipid profiles, and diabetes over the long term [5].

Sri Lanka’s total population is 21.4 million. Adolescents (10- 19 years) comprise 16% of the total population. Out of this 71% of the adolescents are school going and 21.9% of them are non-school going [7]. There are approximately 4 million children studying in 10,000 schools across Sri Lanka [7]. Sri Lanka provides primary and secondary prevention programs for the physical and mental health of students. From 1983 until 2009, Sri Lanka was engaged in a civil conflict which displaced approximately 800,000 people [8]. This conflict had a significant impact on individual health along with disrupting family and community structures resulting in collective trauma [8]. A previous study on mental health issues of 13-15 year-old school students in the Jaffna district revealed that 30% of the students had mental health issues [9]. This is three times higher than the rest of the country, as well as when compared to other South East Asian countries [9]. Currently, within the school system in Northern Sri Lanka there are no guidelines for how to handle behavioural issues in adolescents. The present best practice to is to refer adolescents with behavioural issues to a psychiatrist through their local primary care physician or pediatrician.

Yoga is described in Patanjali’s Yoga Sutras of 900 B. C and originated in India. The meaning of Yoga in Sanskrit language is yoke or bridge. Yoga unites the body and mind or man and God. The umbrella term yoga includes 1) different physical postures (*asanas*), 2) breathing control (*pranayamas),* 3) deep relaxation (*yoganidra)* 4) body cleansing techniques (*kriyas)* and 5) meditation. A study found significant reduction in tension and anxiety after 30 min of yoga 2-3 times per week for 11 weeks in children and adolescents [10]. It also had significant impact on self-esteem, better memory performance, anger control, reduced fatigue and inertia [11].

Yoga taught in schools in other settings has shown improved physical and mental wellbeing of the young people by down regulation of the hypothalamic-pituitary-adrenal axis and sympathetic nervous system [12]. This helped reduce stress and improves self-regulation skills, self- confidence and self-awareness [12]. Physiologically slowing breathing reduces the sympathetic drive thereby reducing anxiety through a feedback mechanism the parasympathetic nervous system gets activated inducing a calm and relaxed state of mind [13]. This effect is immediate as well as sustainable therefore, it is a significantly helpful tool to regulate emotional, social and spiritual wellbeing in practicing adolescents [13]. Currently, yoga is not part of policy or practice within the school system in Northern Sri Lanka.

This study, then built on previous research to develop and implement a yoga-based intervention package to address behavioural issues in school-going adolescents in the post-conflict region of Jaffna, Sri Lanka.

## Methods

This study was a non-randomized controlled trial carried out in 4 educational zones in Jaffna, Northern Province, Sri Lanka over a 6-month period.

The sampling frame was determined using information from the Northern Province Education Ministry website. There are 5 educational zones in Jaffna district. The Island zone consists of an area where the socioeconomic situation and family support is poor, however the other 4 zones are equal in socioeconomic and family support. Therefore, to reduce bias the Island zone was not included. The sample size was calculated using the following formula [14]. The number of participants in this study per cluster was set as (m) = 15

$$\mathrm{N}=\frac{2{\left({\mathrm{Z}}_{a/2}+{\mathrm{Z}}_b\right)}^2\left({s}^2\right)1+\left(\mathrm{m}-1\right)r\Big)}{{\left({m}_1-{m}_2\right)}^2}$$

The calculated sample size for each arm was 331 grade 8 students (ages 13-15). After adding 10% for possible non-responders the final sample was 365 in each arm. As per the cluster size of 15, 25 clusters were randomly selected: 11 schools for each arm in the designated educational zones were recruited for the study. 584 students were included in the intervention arm and 499 in the control group. Vadamaradchi and Valikamum educational zones schools were selected as controls as they were not exposed to any type of regular yoga-based program over the past 2 years, and geographic proximity. Jaffna and Thenmaradchi zones were selected for the yoga-based intervention due to geographic proximity. Within each school allocated to intervention, a grade 8 class (cluster) was randomly selected to be in the intervention group. Within each school allocated to control, a grade 8 (cluster) was randomly selected to be in the control. Students within the clusters were not randomized.

Inclusion criteria for both arms: grade 8 school students (13-14 years old) studying in Jaffna and Thenmaradchi educational zones for 6 months (intervention group) or studying in the Valikamam and Vadamaradchi educational zones for 6 months (control group), without any diagnosed mental health disorders or spine abnormalities. Exclusion criteria for both arms: cognitive or learning impairment, or mental health issue so severe consent could not be gained.

The yoga-based intervention package was developed with a panel of experts including a consultant psychiatrist, religious leaders, consultant community physicians, and an official from the Ministry of Education. It was determined the yoga-based intervention would be delivered in the selected schools from 7:30 am to 7:50 am, Monday to Thursday for 6 months (2 school terms). Below is a detailed description of the yoga-based intervention.

1. Two types of slow breathing techniques (Suga- pranayama (normal deep breathing), Nadi sudhi (alternate nostril) pranayama) for 5-6 min.
2. Surya-namaskaram for 6-8 min.
3. Mindfulness meditation for 5-6 min.

Students in the control group continued with their normal school “keep fit” physical exercise routine. This “keep fit routine” consists of 20 min of dancing to music Mondays to Thursdays across all schools. Those in the intervention group participated in the yoga-based package while those in the controls remained in their “keep fit” routine classes.

The yoga-based intervention was delivered by local yoga teachers who were hired for the first 2 weeks of the intervention. To increase sustainability after the 2-week period student leaders took over the program under supervision of the sports science teachers or counselling teachers.

The Strengths and Difficulties Questionnaire (SDQ) was used pre/post for both groups and had already been validated for use [15]. The SDQ was chosen as it is a brief behavioural screening questionnaire for 3–16-year-olds. The SDQ consists of 25 items divided between 5 scales: emotional symptoms, conduct problems, hyperactivity/inattention, peer relationship problems, and prosocial behaviour. Scores on each scale are added together to generate a total difficulties score (based on 20 items). The SDQ used to quantify baseline behaviour of school-going adolescent participants from respective perspectives of students, parents and teachers.

Assessments were conducted at baseline and at 6 months post yoga intervention for the intervention arm and the control arm. The SDQ was administered to students face-to-face, while teachers and parents completed in their own time. It takes 5-10 min to complete the SDQ. Pre-test questionnaire data collection took 2 weeks and post-test questionnaire data collection took 3 weeks (due to students forgetting to return their parents assessments from home).

At 6 months post-baseline, two focus group discussions were carried with participants in the intervention arm out in two selected educational zones [Jaffna Hindu Ladies College (*n* = 16) and Chavakatcheri Ladies College (*n* = 20)] to explore participant opinions on the intervention program. Focus group were used to deepen understanding of the impact the intervention had. Example questions included, “How do you feel about your educational achievements now?” and “have you noticed any changes in dynamics with your family?” Focus groups lasted 40-45 min each and were run over 2 consecutive days.

A pilot study was carried out for 2 weeks in a different district within Jaffna, Northern Province to identify and overcome any technical issues in the intervention package. Revisions were made as necessary before implementing the full study. The full study was conducted from January-July 2018 in four educational zones of Jaffna, Northern Province, Sri Lanka.

### Primary outcomes

1) Parent and teacher assessments of externalizing problems (conduct problem, hyperactivity) and internalizing problems (emotional issues, peer problems), total difficulty scores and prosocial social scores as measured by the SDQ in both intervention and control groups.

2) Student self-assessments of externalizing problems (conduct problem, hyperactivity) and internalizing problems (emotional issues, peer problems), total difficulty scores and prosocial social scores as measured by the SDQ in both intervention and control groups.

3). Focus group with students in the intervention group who wished to participate to qualitatively investigate any changes in health, school achievement, and family dynamics.

### Analysis

Paired t-test and independent t-test were completed for both arms of the study population using SPSS21. A comparison of post-test scores between the intervention and control arms was completed to understand any significant intervention effect differences. Compliance with the implementation of yoga-based intervention was monitored by the designated teachers in the schools as well as by the principal investigator by a record system. Focus groups were analysed using thematic analysis to understand common themes across the intervention group.

### Ethics approval

Ethical approval for the study was obtained from the Ethics review committee of the Faculty of Medicine, University of Kelaniya (P/217/08/2017). The trial was registered with the Sri Lanka trial registry (Registration No: SLCTR/ 2018/002 Date of Registration: 23rd January 2018). Parents indicated written consent for students, and students indicated assent to participate in the study.

## Results

### Sociodemographic characteristics

There were 535 male, 549 female (*N* = 1084) students in the study. 1056 students were 13 years of age and 28 were 14 years old. 1065 of the students were of Tamil ethnicity, with 19 identifying as non-Tamil. 933 students were of Hindu faith, 132 Christian, and 19 Islamic. There was no significant difference in the age and sex distribution in the intervention and control arms (Table 1).

**Table 1:** Demographic characteristics of children in the yoga intervention trial

Variable	Control (*n* = 499)(%)	Intervention (*N* = 585)(%)
*Sex*		
Male	246 (49.3)	289 (49.4)
Female	253 (50.7)	296 (50.6)
*Age*		
13	488 (97.8)	568 (97.1)
14	11 (2.2)	17 (2.9)
*Ethnic group*		
Tamil	490 (98.2)	575 (98.3)
Others	09 (1.8)	10 (1.7)
*Religion*		
Hindu	429 (86)	504 (86.2)
Christian	61 (12.2)	71 (12.2)
Islam	09 (1.8)	10 (1.7)

**Fig. 1:**
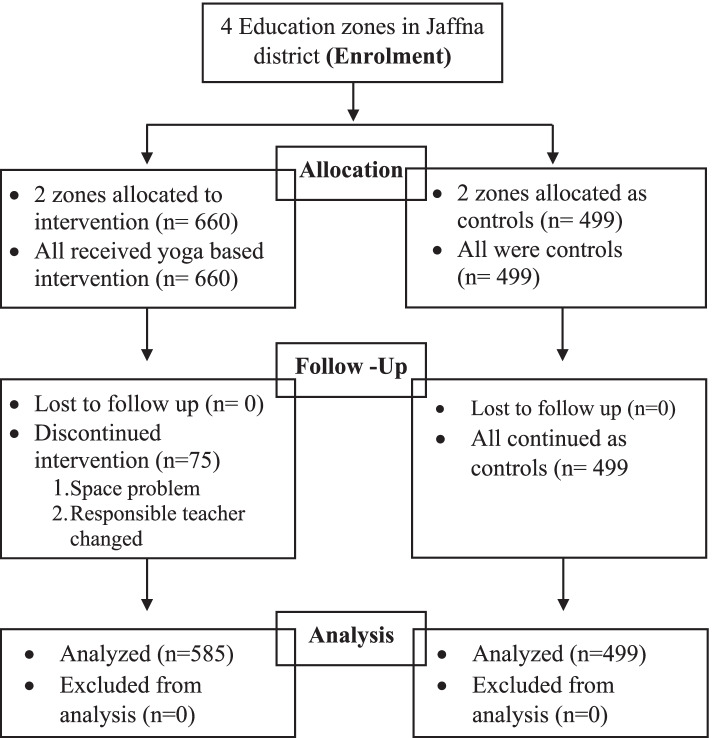
CONSORT Flow diagram of the yoga-based intervention

Despite not matching intervention and control group participants, each group was very similar to each other in regards to sociodemographics (49.3% male in control, 49.4% male in intervention; 50.7 female in control; 50.6% female in intervention). This pattern of similarity extended to ethnicity, age, and religion (Table 1). There was no loss to follow-up in either participant groups over the 6-month time period (Fig. 1).

### Intervention group results

Baseline and the post-intervention SDQ scores from parents, teachers and students were analysed using paired t-tests (Table 2). The parent assessment indicated students’ total difficulty score, as well as emotional conduct, hyperactivity and peer problems reduced after 6 months of participating in the yoga-based intervention, the reduction was highly significant. Student’s prosocial score also significantly increased after 6 months of the yoga-based intervention.

**Table 2:** Comparison of pre- and post-test mean SDQ scores for the intervention arm

SDQ scales and sub scales	Pre- intervention Mean (SD)	Post- intervention Mean (SD)	t- statistic	*p*- value
*Parent*				
Externalizing behaviours	14.5 (2.5)	8.4 (2.6)	41.18	0.001
Conduct problems	7.0 (1.7)	3.7 (1.7)	33.37	0.001
Hyperactivity	7.47 (1.6)	4.7 (1.7)	28.08	0.001
Internalizing behaviours	10.8 (4.6)	5.4 (3.2)	33.12	0.001
Emotional problems	5.3 (2.9)	3.6 (2.1)	11.41	0.001
Peer problems	5.4 (2.1)	4.6 (2.1)	6.71	0.001
Total difficulty score	25.3 (5.7)	16.6 (4.7)	28.12	0.001
Pro social score	2.1 (1.5)	5.38 (2.1)	−30.16	0.001
*Teacher*				
Externalizing behaviours	13.6 (2.5)	9.6 (1.9)	25.70	0.001
Conduct problems	6.4 (1.6)	4.4 (1.4)	20.49	0.001
Hyperactivity	7.2 (1.7)	5.3 (1.6)	17.48	0.001
Internalizing behaviours	12.0 (3.4)	1.52 (2.9)	10.13	0.001
Emotional problems	5.3 (2.9)	5.1 (2.1)	1.55	0.120
Peer problems	7.3 (1.6)	5.4 (1.7)	16.87	0.001
Total difficulty score	26.2 (4.9)	20.1 (4.2)	19.87	0.001
Pro social score	2.3 (1.5)	5.5 (1.8)	−28.49	0.001
*Student*				
Externalizing behaviours	13.8 (2.7)	7.39 (4.7)	26.40	0.001
Conduct problems	7.4 (1.6)	3.1 (2.5)	31.94	0.001
Hyperactivity	6.4 (2.0)	4.27 (2.7)	13.66	0.001
Internalizing behaviours	13.6 (3.3)	10.65 (4.6)	11.37	0.001
Emotional problems	6.5 (2.2)	5.40 (2.9)	6.38	0.001
Peer problems	7.1 (1.9)	5.3 (2.1)	14.43	0.001
Total difficulty score	27.4 (4.9)	18.05 (8.5)	21.16	0.001
Pro social score	2.1 (1.4)	7.3 (2.6)	−36.51	0.001

Parents’ assessments indicate conduct problems and hyperactivity problems were reduced and this was statistically significant [conduct problems *t* (585) = 33.37, *p* = 0.001; hyperactivity problems *t*(584) = 28.08, *p* = 0.001]. Similarly, scores for internalizing problems also reduced and this was statistically significant [emotional problems *t*(584) = 11.41, *p* = 0.001; peer problems *t*(584) = 6.71, *p* = 0.001]. The total difficulty scores also reduced after the intervention and this was statistically significant [*t*(584) = 28.12, *p* = 0.001]. The prosocial score was improved and this was statistically significant [*t*(584) = − 30.16, *p* = 0.001].

Teachers’ assessments for externalizing problems, namely conduct problem and hyperactivity problems were reduced and this was statistically significant [conduct problem *t*(584) = 20.5, *p* = 0.001; hyperactivity problems *t*(584) = 17.5, *p* = 0.001]. Similarly, scores for internalizing problems were reduced but the reduction was statistically significant only for peer problems (emotional problems *t*(584) = 1.6, *p* = 0.12; peer problems *t*(584) = 16.9, *p* = 0.001]. Total difficulty scores reduced after the intervention and this was statistically significant [*t*(584) = 19.9, *p* = 0.001). The prosocial score improvement was also statistically significant [*t*(584) = − 28.5, *p* = 0.001).

Students’ self-assessment for externalizing problems, namely conduct problem and hyperactivity problems were reduced and this was statistically significant [conduct problems *t*(584) = 31.9, *p* = 0.001; hyperactivity problems *t*(584) = 13.7, *p* = 0.001]. Scores for internalizing problems were reduced and this was statistically significant [emotional problems *t*(584) = 6.4, *p* = 0.001; peer problems *t*(584) = 14.4, *p* = 0.001]. Total difficulty scores also reduced after the intervention and this was statistically significant [*t*(584) = 21.2, *p* = 0.001]. The prosocial score improvement was statistically significant [*t*(584) = − 36.5, *p* = 0.001). Please see Table 2 for more details on intervention group results.

### Control group results

The schools in the control group continued with their routine keep fit exercises and results can be found in Table 3. Pre- and post-assessment scores revealed the total difficulty scores and emotion, conduct, hyperactivity, and peer problems worsened. Results found higher scores for each of the sub scales by parents, teachers and students themselves at the end of the 6-month period, and this was highly significant. Parents’ perspectives increased on conduct problems [*t*(499) = − 25.9, *p* = 0.001], hyperactivity problems [*t*(499) = − 51.4, *p* = 0.001). Similarly, scores for internalizing problems increased and the increase was statistically significant. Emotional problems increased [*t*(499) = − 65.6, *p* = 0.001], along with peer problems [*t*(499) = − 47.6, *p* = 0.001]. Total difficulty scores increased after 6 months in the control group and the increase was statistically significant [*t*(499) = − 80.3, *p* = 0.001]. The prosocial score was reduced and the reduction was also statistically significant [*t*(499) = 43.1, *p* = 0.001].

**Table 3:** Comparison of pre and post-test mean SDQ scores for the control arm

SDQ scales and sub scales	Pre- intervention Mean (SD)	Post- intervention Mean (SD)	t- statistic	***p***- value
*Parent*				
Externalizing behaviours	3.9 (2.2)	11.2 (2.4)	−50.48	0.001
Conduct problems	1.8 (1.3)	4.2 (1.6)	−25.89	0.001
Hyperactivity	2.2 (1.3)	7.0 (1.7)	−51.36	0.001
Internalizing behaviours	3.8 (1.8)	13.8 (2.3)	− 76.41	0.001
Emotional problems	1.8 (0.9)	7.0 (1.6)	−65.58	0.001
Peer problems	2.0 (1.5)	6.8 (1.7)	−47.57	0.001
Total difficulty score	7.8 (3.3)	25.0 (3.6)	−80.31	0.001
Pro social score	8.0 (1.8)	3.9 (1.2)	43.11	0.001
*Teacher*				
Externalizing behaviours	9.9 (2.6)	12.8 (2.2)	−18.58	0.001
Conduct problems	3.7 (1.7)	5.9 (1.5)	−22.46	0.001
Hyperactivity	6.3 (1.6)	6.9 (1.6)	−5.84	0.001
Internalizing behaviours	10.2 (4.1)	13.5 (2.7)	−16.89	0.001
Emotional problems	5.0 (2.9)	6.3 (1.7)	−9.46	0.001
Peer problems	5.1 (1.9)	7.2 (1.7)	−19.17	0.001
Total difficulty score	20.1 (5.6)	26.3 (3.7)	−22.02	0.001
Pro social score	3.8 (1.8)	2.4 (1.5)	13.46	0.001
*Student*				
Externalizing behaviours	7.2 (2.9)	13.5 (2.3)	−38.16	0.001
Conduct problems	3.3 (1.9)	6.6 (1.6)	−30.20	0.001
Hyperactivity	3.9 (1.7)	6.8 (1.6)	−28.49	0.001
Internalizing behaviours	6.6 (3.0)	13.1 (2.4)	−38.08	0.001
Emotional problems	3.6 (1.9)	6.7 (1.7)	−27.14	0.001
Peer problems	3.0 (1.8)	6.4 (1.6)	−31.45	0.001
Total difficulty score	13.8 (4.9)	26.5 (3.7)	−47.91	0.001
Pro social score	6.7 (1.6)	8.1 (1.7)	13.13	0.001

Teachers’ assessment for externalizing problems, namely conduct problems [t(499) = − 22.4, *p* = 0.001] and hyperactivity problems [t(499) = − 5.8, *p* = 0.001] increased significantly. Similarly, scores for internalizing problems such as emotional problems [*t*(499) = − 9.5, *p* = 0.001], and peer problems [*t*(499) = − 19.2, *p* = 0.001]. Total difficulty scores increased post-intervention and this increase was statistically significant [*t*(499) = − 22.0, *p* = 0.001]. The prosocial score was reduced and this was statistically significant [*t*(499) = 13.5, *p* = 0.001].

Students’ self-assessment for externalizing problems, such as conduct problems [*t*(499) = − 30.2, *p* = 0.001], and hyperactivity problems [*t*(499) = − 28.5, *p* = 0.001] increased significantly. Similarly, scores for internalizing problems such as emotional problems [*t*(499) = − 27.1, *p* = 0.001], and peer problems [*t*(499) = − 31.4, *p* = 0.001] increased significantly. Total difficulty scores increased significantly post-intervention [*t*(499) = − 47.9, *p* = 0.001]. The prosocial score also increased significantly [*t*(499) = 13.1, *p* = 0.001].

### Comparison of intervention and controls

A statistically significant difference can be seen between the two groups in the post-test (Table 4). It should be noted the control group had consistently lower scores at baseline.

**Table 4:** Comparison of post-test mean SDQ scores between the intervention and control groups

SDQ scales and sub scales	Post- intervention Mean (SD)	Post- Control Mean (SD)	t- statistic	*p*- value
*Parent*				
Externalizing behaviour	8.02 (3.23)	5.49 (3.14)	13.16	0.001
Conduct problems	3.44 (1.92)	2.12 (1.79)	11.71	0.001
Hyperactivity	4.59 (1.96)	3.32 (1.77)	10.75	0.001
Internalizing behaviour	8.05 (3.29)	5.51 (3.14)	13.03	0.001
Emotional problems	3.5 (2.09)	3.02 (2.01)	4.18	0.001
Peer problems	4.50 (2.17)	2.49 (1.76)	16.72	0.001
Total difficulty score	16.07 (5.75)	11.01 (5.52)	14.89	0.001
Pro social score	6.12 (2.75)	8.15 (1.73)	−14.47	0.001
*Teacher*				
Externalizing behaviour	8.03 (3.44)	5.27 (3.29)	13.59	0.001
Conduct problems	3.45 (2.04)	2.00 (1.79)	12.43	0.001
Hyperactivity	4.59 (2.07)	3.27 (1.95)	10.90	0.001
Internalizing behaviour	8.64 (3.99)	5.10 (3.25)	16.03	0.001
Emotional problems	4.01 (2.54)	2.8 (2.12)	8.22	0.001
Peer problems	4.63 (1.99)	2.26 (1.78)	20.75	0.001
Total difficulty score	16.66 (6.93)	10.37 (5.84)	16.22	0.001
Pro social score	6.63 (2.22)	8.-7 (2.02)	−15.93	0.001
*Student*				
Externalizing behaviour	6.27 (4.26)	5.51 (3.31)	3.13	0.002
Conduct problems	2.72 (2.19)	2.53 (1.84)	1.49	0.135
Hyperactivity	3.55 (2.54)	2.98 (1.93)	3.95	0.001
Internalizing behaviour	8.65 (4.75)	5.85 (3.36)	10.3	0.001
Emotional problems	4.50 (2.83)	3.42 (2.12)	6.89	0.001
Peer problems	4.15 (2.40)	2.43 (1.85)	12.55	0.001
Total difficulty score	14.92 (8.33)	11.63 (5.88)	7.80	0.001
Pro social score	7.66 (2.35)	8.21 (1.65)	−4.28	0.001

Between groups parent post-test assessment of externalizing behaviour showed a significant increase in conduct problems [*t* (557) = 11.71, *p* = 0.001] and hyperactivity [*t* (557) = 10.75, *p* = 0.001]. Parent post-test assessment of emotional problems [*t* (557) = 4.18, *p* = 0.001] and peer problems [*t* (557) = 16.72, *p* = 001] increased significantly. Total difficulty scores also increased [*t* (557 = 14.89, *p* = 0.001], while pro social scores decreased [*t* (557) = − 14.47, *p* = 0.001].

Between groups teacher post-assessment of externalizing behaviour showed a significant increase in conduct problems [*t* (557) = 12.43, *p* = 0.001], and hyperactivity [*t* (557) = 10.90, *p* = 0.001]. Internalizing behaviours such as emotional problems also increased [*t* (557) = 8.22, *p* = 0.001], along with peer problems [*t* (557) = 20.75, *p* = 0.001]. Total difficult scores also increased [*t* (557) = 16.22, *p* = 0.001] while pro social scores decreased [*t* (557) = − 15.93, *p* = 0.001].

Between group results from students’ assessments found externalizing behaviours such as conduct problems [*t* (557) = 3.13, *p* = 0.001] and hyperactivity [*t* (557) = 1.49, *p* = 0.001] increased. Internalizing behaviours such as emotional problems increased [*t* (557) = 6.89, *p* = 0.001] along with peer problems [*t* (557) = 12.55, *p* = 0.001]. Total difficulty scores increased [*t* (557) = 7.80, *p* = 0.001] while pro social scores decreased [*t* (557) = − 4.28, *p* = 0.001].

### Focus group results

Results found from the focus groups found students found the yoga program had a positive impact on their health, school achievements and family dynamics. A majority of students noted they felt their stress levels had been significantly reduced, with one student noting, “my mind has quieted a lot now”. Students also noted they felt, “problem solving is relatively easy for me now” and reported feeling less fatigued, “I used to get tired after 3^rd^ subject in school but now I am motivated to learn until the last period of school.” Students also noted a significant reduction in asthmatic attacks, “after this program I have not visited the hospital for nebulization.” General health also appeared to improve post-intervention with one student noting, “complaints about body pains have reduce quite a lot now”. Students also shared that they felt more confident noting, “I can quickly complete my homework easily.” Finally, students noted they felt their ability to handle family dynamics had improved, “I am able to tolerate others views without heated argument”, and “I have reduced fighting with my siblings now”.

## Discussion

The current study used a cluster, non-randomized study to evaluate the implementation of a yoga-based intervention package in grade 8 school children (early adolescents) to address behavioural problems. The study was conducted in the post-conflict region of Jaffna, Sri Lanka. The period of adolescence can be difficult both physically and psychologically. Issues can be compounded in an area that has been exposed to conflict and trauma [8, 9]. Behavioural issues in school-going adolescents can go unnoticed by parents and teachers and if left unidentified or untreated can lead to negative health consequences [5]. The use of yoga within school settings can help to address behavioural issues and reduce risk of future health issues. Practicing Surya-namaskaram, breathing control techniques and mindfulness meditation reduces both externalizing symptoms (conduct problems and hyperactivity) as well as internalizing symptom (emotional problem and peer problems).

Our results indicate that within the intervention group there was a significant positive impact of yoga on externalizing behaviours (conduct problems, hyperactivity) and internalizing behaviours (emotional problems, peer problems) when assessed by parents, teachers and students. Prosocial scores (good behaviour) also increased significantly in the intervention arm and total difficult scores reduced across all assessments. The control group findings indicate the routine ‘keep fit’ exercises routinely engaged with at school sites did not have any significant impact on externalizing or internalizing behaviours. It needs to be noted that comparison of post-test scores between groups did not indicate any meaningful effect. This could be due to lower observed baseline control group scores or could be due to control and intervention groups not being matched from the outset. The effect size of the intervention could not be calculated as the linearity and homogeneity assumptions for Analysis of covariates (ANCOVA) could not be fulfilled. This does limit our results, however our findings within the intervention group are still important to report.

Our findings agree with findings of several other similar studies. A systematic review revealed pre- and post-yoga intervention scores in children and adolescents aged 5–18 years old showed significant reduction in their tension and anxiety after performing 30 min of yoga practice, 2-3 times per week for 11 weeks [11]. The children demonstrated greater self-esteem and better memory performance. Whereas the controls continued to worsen over time [11]. Mindfulness has also been shown to promote psychological health and well-being in children and adolescents [16]. In an urban school in the U. S, 37 children who were identified as having emotional and behavioural disorders (EBD) were offered yoga training for 3 ½ months [17]. The findings of pre- and post- intervention assessments by the students’ parents and their teachers suggested that children who were trained in yoga improved their attention in class and adaptive skills, and their depressive and behavioural symptoms were reduced significantly [17]. A meta-analysis suggests that yoga taught in schools improves physical and mental wellbeing of the young people by down-regulating the Hypothalamic-Pituitary-Adrenal axis (HPA) and the sympathetic nervous system. This physiological change improves their emotional balance, mood, resilience and their self-regulation skills [12]. An 8-week mindfulness-based stress reduction programme in a U. S medical school revealed that the meditators brain had an increased concentration of grey matter in the hippocampal area (centre for learning, memory and emotional regulation), and a reduced concentration of grey matter in the amygdala (centre for fear, anxiety and stress) [18] of an individual when compared to the controls.

This study found the practice of yoga (asana) and breath control improves attention and reduces behavioural symptoms in school-going children. Results indicate this intervention had a positive effect on student health, confidence, school achievement, and improved family dynamics. It is recommended this program be scaled up across Sri Lanka to reach all school-going children across the country. It is also recommended that this type of intervention be implemented in other, similar post-conflict settings.

### Strengths and limitations

To reduce contamination, two education zones that were geographically close were selected as controls and the other two selected as trial zones. Schools within the control and trial zones were selected randomly minimizing selection bias. Although yoga teachers were employed in the first 2 weeks, the students took leadership on a roster basis and continued over the 6-month period making the program cost-efficient and sustainable.

To reduce overall bias rigorous criteria were used for participant selection, the four education zones were chosen to ensure participants in both intervention and control groups originated from similar general population, and validated assessment instruments were used. Bias was reduced in the analysis as the data set was complete and there were no outliers found.

Urban, rural schools or grade 1AB (with Advanced level Science included), 1C (Advanced level Arts Commerce included) and 1C (schools with only up to Ordinary level (grade 11) schools were not compared separately during the analysis and there could have been significant differences missed. Intervention and control groups were not randomized and researchers were not blind to group allocation which could have introduced selection bias.

Focus group discussions were only conducted with the intervention group, if focus groups were also conducted with control groups important information on current school exercise programs could have been gained. Focus groups were also quite large (*n* = 16; *n* = 20) and this may have limited people’s ability to share observations and insights. Further, both groups were female only which limits generalizability.

### Recommendations for future research

Future research should include the deprived Island educational zone as there may be specific student behavioural issues to address in this region. Further, findings from urban, rural schools or grade 1AB (with Advanced level Science included), 1C (Advanced level Arts Commerce included) and 1C (schools with only up to Ordinary level (grade 11) schools should be compared separately to understand any significant differences. Focus group work should be completed with smaller numbers of participants and with a more generalizable sample. Future research should also include scaling up implementation of the yoga-based intervention across other regions of the country to address potential unmet need within the school system.

The comparison of post-test scores between intervention and control groups was not found to be meaningful perhaps due to lower baseline scores of the control group or because the groups were not matched from the outset. Further, the effect size could not be calculated as the linearity and homogeneity assumptions for ANCOVA could not be fulfilled. Future research should address these important limitations regarding effect size.

## Conclusions

This school-based yoga program appears to help to reduce negative behaviours and promote positive behaviours in school-going children in the post-conflict region of Jaffna, Sri Lanka. This intervention helped equip children to develop healthy coping skills to reduce risk of behavioural problems and negative health consequences in the future. This intervention is low-cost, simple to implement, and could be an effective strategy for school-going children in similar post-conflict situations.

## Data Availability

The dataset analysed during the current study are available from the corresponding author on reasonable request.

## Abbreviations

WHO: World Health Organization
HIV/AIDS: Human Immuno Defficiency Virus/Aquired Immuno Deficiency Syndrome
CDC: Centre for Disease Control
STDs: Sexually transmitted diseases
SDQ: Strengths and Difficulties Questionnaire
SPSS21: Statistical product and service solutions
SLCTR: Sri Lanka trial registry
ANCOVA: Analysis of covariates
EBD: Emotional and behavioural disorders
HPA: Hypothalamic-Pituitary-Adrenal axis
MRI: Medical Research Institute
